# Supplementary material for: The effect of variant interference on de novo assembly for viral deep sequencing
Source: BMC Genomics. 2020 Jun 22;21:421. doi: 10.1186/s12864-020-06801-w (PMC7306937; doi:10.1186/s12864-020-06801-w)
Supplement: Supplementary file 1 — Additional file 1. Supplemental Text: Analysis of viral GenBank records demonstrated the advent of NGS in viral sequencing in the last two decades. Supplement Figure S1. Workflow diagrams of simulated data from data creation through de novo assembly. Supplement Figure S2. Analysis of the final contig assembly graphs for a clinical sample containing enterovirus A71 (EV-A71) variants using Bandage. Supplement Figure S3. Assembly with three simulated variants. Supplement Table S1. Total counts from NCBI’s GenBank non-redundant nucleotide database. Supplement Table S2. Total count of sequencing technologies for sequences >2000 nt in the NCBI GenBank non-redundant nucleotide database for years 2012–2019. Supplement Table S3. Total counts from NCBI’s GenBank non-redundant nucleotide database with multiple sequencing technologies listed per entry. Supplement Table S4. Total counts from NCBI’s GenBank non-redundant nucleotide database of all entries with three and four sequencing technologies listed. Supplement Table S5. Total count of assembly programs used to generate sequences >2000 nt in the NCBI GenBank non-redundant nucleotide database. Supplement Table S6. The 10 de novo assemblers used for analysis of the simulated data, as categorized by their underlying assembly algorithms. [file 12864_2020_6801_MOESM1_ESM.docx]

**Supplemental Information**

**Analysis of viral GenBank records**

**The advent of NGS fuels viral sequencing**

As of December 2019, GenBank’s non-redundant nucleotide database had grown to more than 2.7 million virus sequences, with the annual number of new sequences deposited increasing by 880% between 2000 and 2019 [Figure 1a and Supplement Table S1]. GenBank entries started incorporating information on the sequencing technology platform used in 2011. Through 2019, 182,045 viral entries (27%) had documented utilization of NGS sequencing technology, compared to 665,783 entries (73%) utilizing Sanger methods [Figure 1b and Supplement Table S1]. Illumina was the most common NGS platform used for viral sequencing since 2014, with approximately 80,000 more total entries as compared to the next most popular NGS platform (454) [Figure 1d & e]. Although NGS usage has risen tremendously, Sanger sequencing still contributed the majority of all viral sequences. This is likely because Sanger is still attractive for generating short viral sequences over genotyping windows or other informative regions. If only long sequences (≥2000 nt) are considered, NGS technologies surpassed Sanger as the dominant strategy for sequencing in 2017 [Figure 1f and Supplement Table S2], with the same trend continuing for 2018 and 2019.

A total of 27,21190 counts of sequencing technologies were listed for the long (>2000 nt) viral GenBank entries in 2019. NGS technologies were listed in 65.1% (17,690/27,190) of entries, versus 34.9% of entries (9500/27,190) for Sanger. Illumina was identified as the most dominant NGS technology, accounting for 16,045/17,690 entries (90.7%) [Figure 1g and Supplement Table S2].

Multiple sequencing technologies may be used to generate viral sequence for one entry. The most common combination observed was 454 and Sanger (18,124 entries), likely due to the early emergence of the 454 technology compared to other NGS platforms [Figure 1c and Supplement Table S3]. This is followed by Illumina and Sanger (11,587), Illumina and 454 (3,388), Illumina and Ion Torrent (3,044), and Illumina and PacBio (1,054). Interestingly, more recently released longer-read platforms like PacBio and Oxford Nanopore tended to be paired with Illumina more frequently compared to traditional Sanger sequencing. A small number of studies even combined three or four different sequencing technologies (626 and 6 entries, respectively) [Supplement Table S4]. Some users employed a combined approach to circumvent the inherent flaws of one sequencing platform, particularly for genome finishing.[1] For example, after NGS has been used to generate the majority of a RNA virus genome, RACE (Rapid amplification of cDNA ends) is typically performed with Sanger to obtain the 5’ or 3’ termini.[2, 3]

***De novo* assembly plays a major role in analyzing long viral sequences**

We analyzed the assembly methods used for GenBank entries of long sequences (≥2000 nt) from 2012 to 2019 when NGS usage become relevant [Figure 1h & i and Supplement Table S5]. The number of programs used to assemble viral sequences has steadily increased over time. With new sequencing technologies emerging and computational power continually improving, the development of new and better assembly programs always follows suite. The use of specifically-designed *de novo* assembly programs (ABySS, BWA, Canu, Cap3, IDBA, MIRA, Newbler, SOAPdenovo, SPAdes, Trinity, and Velvet) has increased from less than 1% of viral sequence entries in 2012, to 20% of all viral sequence entries in 2019. A similar increase was observed for reference-mapping software (i.e., Bowtie and Bowtie2), from 0.03% in 2012 to 12.5% in 2019. Multifunctional programs that offer both assembly options, including CLC Genomics Workbench (CLC), DNA Baser, DNASTAR, Geneious, and Sequencher, were by far the most popular option for the years 2013-2019. However, since these commercial software packages can perform both *de novo* and reference-mapping assembly, the exact sequence assembly strategy used for these records is unknown, and thus the contributions of both *de novo* assembly and reference recruitment are likely underestimated.

Reference

1. Phillippy AM: **New advances in sequence assembly**. *Genome research* 2017, **27**(5):xi-xiii.

2. Olivarius S, Plessy C, Carninci P: **High-throughput verification of transcriptional starting sites by Deep-RACE**. *BioTechniques* 2009, **46**(2):130-132.

3. Lagarde J, Uszczynska-Ratajczak B, Santoyo-Lopez J, Gonzalez JM, Tapanari E, Mudge JM, Steward CA, Wilming L, Tanzer A, Howald C *et al*: **Extension of human lncRNA transcripts by RACE coupled with long-read high-throughput sequencing (RACE-Seq)**. *Nature Communications* 2016, **7**:12339.

**
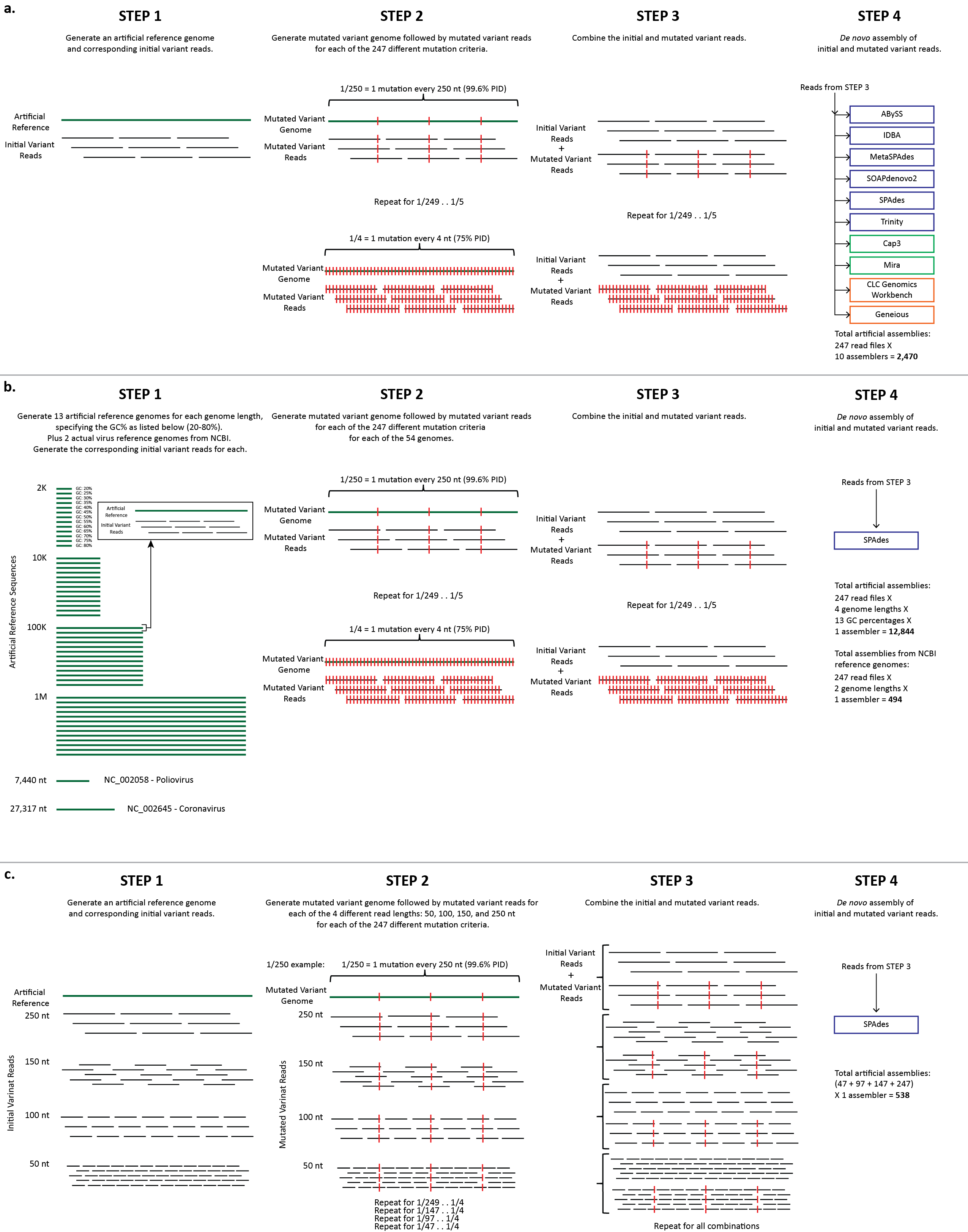
**

**Supplement Figure S1. Workflow diagrams of simulated data from data creation through *de novo* assembly.**

**(a)** **Comparison of assemblers.** First, an artificial reference genome and corresponding initial variant reads were created with the following constraints: (1) reference genome length: 100K; (2) GC% of reference genome: 50%; (3) read length: 250 nt; and (4) coverage: 50X. Second, an artificial mutated variant genome and corresponding mutated variant reads were created 247 times, each with a differing pairwise percent identity ranging from 1 mutation every 4 nucleotides (75% PID) to 1 mutation in every 250 nucleotides (99.6% PID). The initial and mutated variants were then combined and used as input for 10 different *de novo* assemblers with varying underlying algorithms. A total of 2,470 assemblies were performed. **(b)** **Comparison of genome length and GC%.** First, 13 artificial reference genomes and corresponding initial variant reads were created for four different genome lengths (2Kb, 10Kb, 100Kb, and 1Mb), each specifying a different GC% ranging from 20%–80%. In addition, two actual virus reference genomes from NCBI were included, NC_002058 and NC_002645, with genome lengths of 7,440 nt and 27,317 nt, respectively. Read lengths of 250 nt with a coverage of 50X were used for all genomes. Second, an artificial mutated variant genome and corresponding mutated variant reads were created 247 time, each with a differing pairwise percent identity ranging from 1 mutation every 4 nucleotides (75% PID) to 1 mutation in every 250 nucleotides (99.6% PID). The initial and mutated variants were then combined for each and used as input for the SPAdes *de novo* assembler. A total of 13,338 assemblies were performed. **(c) Comparison of read length.** First, an artificial reference genome and corresponding initial variant reads were created with the following constraints: (1) reference genome length: 100K; (2) GC% of reference genome: 50%; (3) read lengths: 50 nt, 100 nt, 150 nt, or 250 nt; and (4) coverage: 50X. Second, an artificial mutated variant genome and corresponding mutated variant reads were created, each with a differing pairwise percent identity ranging from 1 mutation every 4 nucleotides (75% PID) up to 1 mutation in every 250 nucleotides (99.6% PID). The initial and mutated variants created for each of the four read lengths were then grouped by read length size and used as input for SPAdes *de novo* assembler. A total of 538 assemblies were performed.


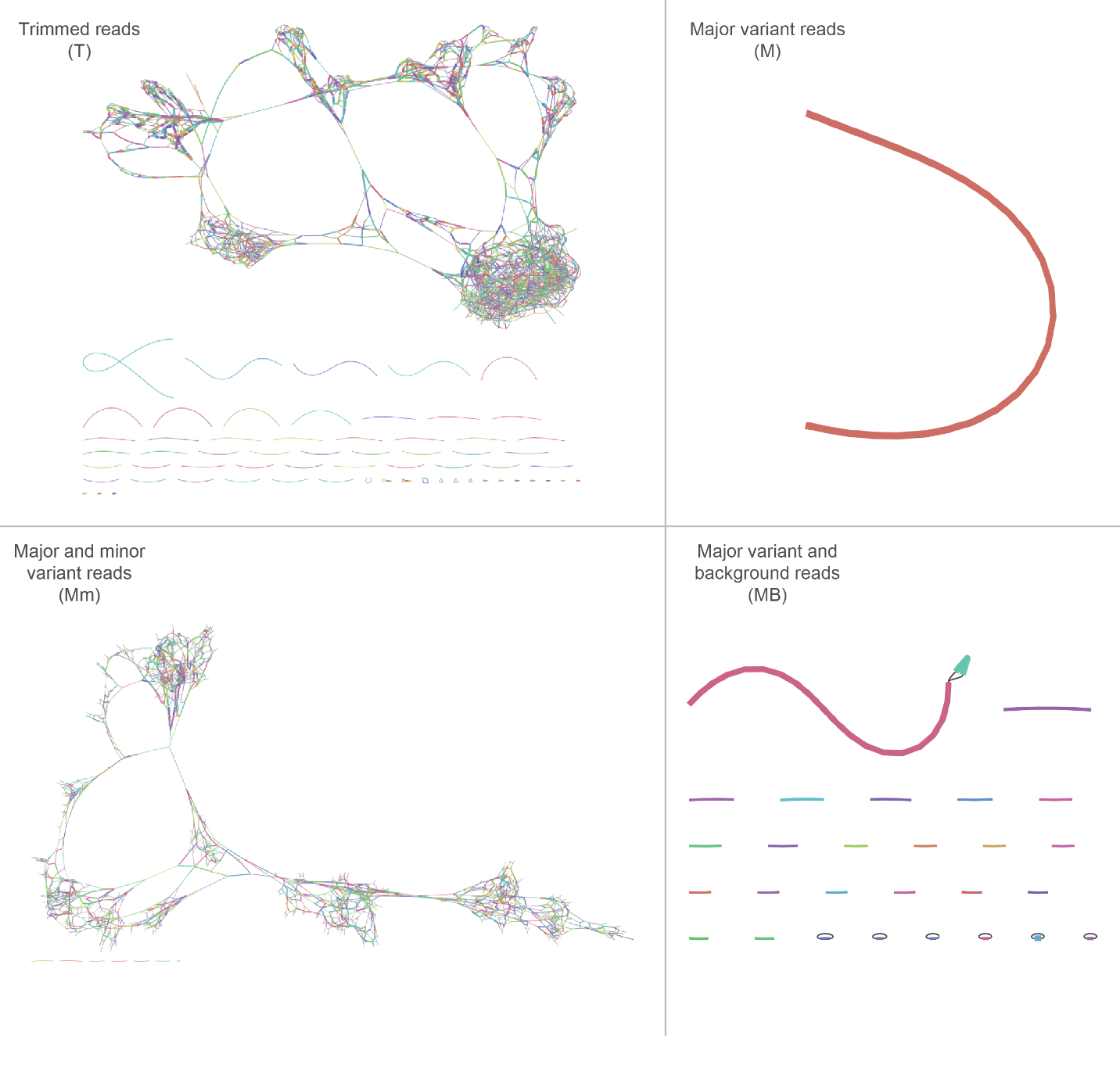


**Supplement Figure S2. Analysis of the final contig assembly graphs for a clinical sample containing enterovirus A71 (EV-A71) variants using Bandage.** Based on the four assemblies in Figure 5, Bandage was used to display the contig graphs from each SPAdes output. The visualizations for T, Mm, and MB show the effects of variant interference, while M shows the ideal assembly.


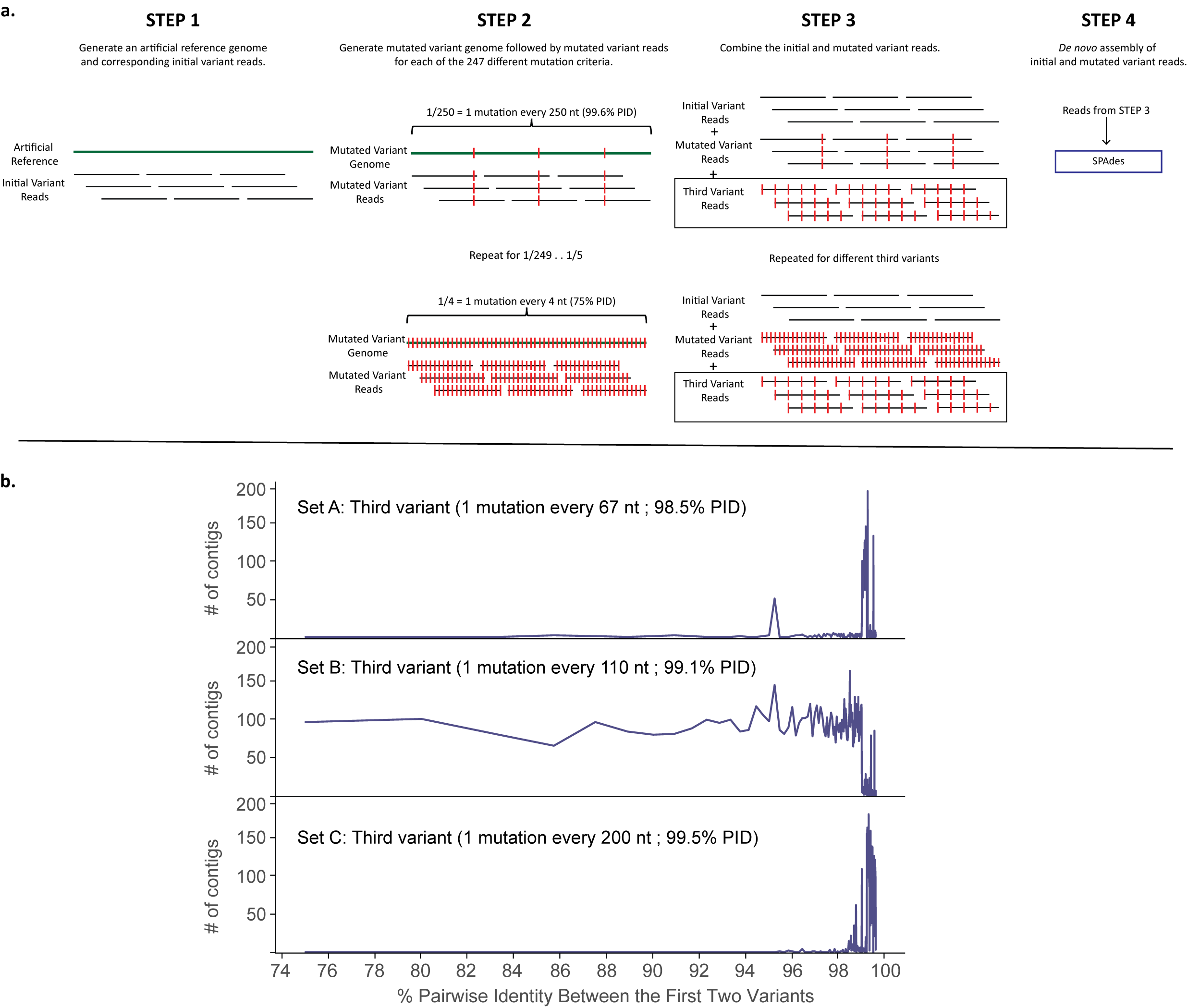


**Supplement Figure S3. Assembly with three simulated variants. (a)** Experimental design was similar to the third experiment as in Fig. 3B with SPAdes, except that a third variant was added. **(b)** The number of contigs generated containing variants differed with a range of percentage identities (PID). X-axis shows the PID between the first two variants. The set A, B, and C show the PID between the first and the third variants. Set A, B, and C are selected to have PID within the thresholds of VD, VI, and VS respectively. Simulation Setting: 50X coverage of reads; pair-end reads; read length 250.

| **Year** | **Total # of viral** | **Total** | **Total** | **Total # of entries with** | **Sequencing Technology Breakdown** | | | | | | | |
| --- | --- | --- | --- | --- | --- | --- | --- | --- | --- | --- | --- | --- |
|  | **entries in GenBank** | **count** | **omitted** | **at least one Seq. Tech.** | **Sanger** | **454** | **Illumina** | **IonTorrent** | **Oxford NP** | **PacBio** | **SOLiD** | **Other** |
| **2019** | 236562 | 244515 | 128071 | 116444 | 76454 | 672 | 36242 | 2255 | 615 | 75 | 1 | 130 |
| **2018** | 218473 | 220018 | 107702 | 112316 | 89297 | 406 | 19175 | 2732 | 90 | 188 | 57 | 371 |
| **2017** | 238367 | 243849 | 108021 | 135828 | 85194 | 15999 | 31279 | 2130 | 46 | 940 | 14 | 226 |
| **2016** | 235477 | 237569 | 107090 | 130479 | 102837 | 2971 | 22185 | 2111 | 119 | 67 |  | 189 |
| **2015** | 197440 | 211177 | 71148 | 140029 | 102440 | 15517 | 17625 | 3048 |  | 6 | 14 | 1379 |
| **2014** | 158579 | 163092 | 66217 | 96875 | 81515 | 5452 | 7399 | 2345 |  | 19 | 30 | 115 |
| **2013** | 198540 | 202232 | 108365 | 93867 | 84527 | 5243 | 2474 | 758 |  | 8 | 61 | 796 |
| **2012** | 172850 | 173324 | 126821 | 46503 | 43509 | 1194 | 277 | 403 |  | 7 | 12 | 1101 |
| **2011** | 181315 | 181319 | 176355 | 4964 | 5 | 4811 | 147 |  |  |  | 1 |  |
| **2010** | 131962 | 131962 | 131960 | 2 | 2 |  |  |  |  |  |  |  |
| **2009** | 213549 | 213549 | 213549 |  |  |  |  |  |  |  |  |  |
| **2008** | 109265 | 109265 | 109265 |  |  |  |  |  |  |  |  |  |
| **2007** | 88996 | 88996 | 88996 |  |  |  |  |  |  |  |  |  |
| **2006** | 94444 | 94444 | 94444 |  |  |  |  |  |  |  |  |  |
| **2005** | 58245 | 58245 | 58245 |  |  |  |  |  |  |  |  |  |
| **2004** | 53841 | 53842 | 53834 | 8 | 1 | 4 | 2 |  |  |  |  | 1 |
| **2003** | 38578 | 38578 | 38576 | 2 | 2 |  |  |  |  |  |  |  |
| **2002** | 33412 | 33412 | 33412 |  |  |  |  |  |  |  |  |  |
| **2001** | 28305 | 28305 | 28304 | 1 |  | 1 |  |  |  |  |  |  |
| **2000** | 26871 | 26871 | 26871 |  |  |  |  |  |  |  |  |  |
| **1999** | 17266 | 17266 | 17266 |  |  |  |  |  |  |  |  |  |
| **1998** | 13840 | 13840 | 13840 |  |  |  |  |  |  |  |  |  |
| **1997** | 12378 | 12378 | 12378 |  |  |  |  |  |  |  |  |  |
| **1996** | 8988 | 8988 | 8987 | 1 |  |  | 1 |  |  |  |  |  |
| **1995** | 7475 | 7475 | 7475 |  |  |  |  |  |  |  |  |  |
| **1994** | 5449 | 5449 | 5449 |  |  |  |  |  |  |  |  |  |
| **1993** | 9185 | 9185 | 9184 | 1 |  |  | 1 |  |  |  |  |  |
| **1992** | 1754 | 1754 | 1754 |  |  |  |  |  |  |  |  |  |
| **1991** | 725 | 725 | 725 |  |  |  |  |  |  |  |  |  |
| **1990** | 364 | 364 | 363 | 1 |  |  | 1 |  |  |  |  |  |
| **1989** | 424 | 424 | 424 |  |  |  |  |  |  |  |  |  |
| **1988** | 269 | 269 | 269 |  |  |  |  |  |  |  |  |  |
| **1987** | 159 | 159 | 159 |  |  |  |  |  |  |  |  |  |
| **1986** | 114 | 114 | 114 |  |  |  |  |  |  |  |  |  |
| **1985** | 130 | 130 | 130 |  |  |  |  |  |  |  |  |  |
| **1984** | 19 | 19 | 19 |  |  |  |  |  |  |  |  |  |
| **1983** | 92 | 92 | 92 |  |  |  |  |  |  |  |  |  |
| **1982** | 108 | 108 | 108 |  |  |  |  |  |  |  |  |  |
| **TOTALS** | **2793810** | **2833303** | **1955982** | **877321** | **665783** | **52270** | **136808** | **15782** | **870** | **1310** | **190** | **4308** |

**Supplement Table S1. Total counts from NCBI’s GenBank non-redundant nucleotide database.**

**†** *Total count* is the combination of all sequencing technologies listed for each entry plus the total number of entries with sequencing technology omitted. This number is higher than the *Total # of viral entries in GenBank* because it accounts for all entries with multiple sequencing technologies listed.

Sequencing Technology, Seq. Tech.; Oxford Nanopore, Oxford NP; Pacific Biosciences, PacBio

| **NGS** | **Year** | | | | | | | |
| --- | --- | --- | --- | --- | --- | --- | --- | --- |
| **Platforms** | **2019** | **2018** | **2017** | **2016** | **2015** | **2014** | **2013** | **2012** |
| **454** | 119 | 222 | 1029 | 634 | 4987 | 1531 | 1642 | 376 |
| **Sanger** | 9500 | 8352 | 12564 | 13571 | 20216 | 14294 | 13646 | 10847 |
| **Illumina** | 16045 | 9542 | 12615 | 12629 | 4121 | 4414 | 1266 | 230 |
| **PacBio** | 74 | 13 | 17 | 67 | 1 | 12 | 1 | 0 |
| **IonTorrent** | 1091 | 1362 | 923 | 1342 | 1217 | 1131 | 408 | 171 |
| **Oxford NP** | 292 | 71 | 46 | 119 | 0 | 0 | 0 | 0 |
| **SOLiD** | 1 | 2 | 8 | 0 | 0 | 13 | 29 | 1 |
| **Other** | 68 | 197 | 15 | 4 | 0 | 5 | 10 | 41 |
| **Helicos** | 0 | 0 | 0 | 0 | 1 | 0 | 0 | 0 |
| **TOTALS** | **27190** | **19761** | **27217** | **28366** | **30543** | **21400** | **17002** | **11666** |

**Supplement Table S2. Total count of sequencing technologies for sequences >2000 nt in the NCBI GenBank non-redundant nucleotide database for years 2012–2019.**These numbers were found with the following search criteria: “viruses,” “genomic RNA/DNA,” “GenBank (No RefSeq),” length: 2000 to 2000000, release date: 1/1/201X to 12/31/201X, and “sequencing technology” in any field.

Oxford Nanopore, Oxford NP; Pacific Biosciences, PacBio

| **Year** | **Total # of entries with** | **Total # of entries with** | **Total # of entries with** |
| --- | --- | --- | --- |
|  | **two Seq. Techs.** | **three Seq. Techs.** | **four Seq. Techs.** |
| **2019** | 7853 | 50 |  |
| **2018** | 1453 | 46 |  |
| **2017** | 5468 | 7 |  |
| **2016** | 2008 | 42 |  |
| **2015** | 13156 | 283 | 5 |
| **2014** | 4457 | 28 |  |
| **2013** | 3409 | 140 | 1 |
| **2012** | 414 | 30 |  |
| **2011** | 4 |  |  |
| **2010** |  |  |  |
| **2009** |  |  |  |
| **2008** |  |  |  |
| **2007** |  |  |  |
| **2006** |  |  |  |
| **2005** |  |  |  |
| **2004** | 1 |  |  |
| **2003** |  |  |  |
| **2002** |  |  |  |
| **2001** |  |  |  |
| **2000** |  |  |  |
| **1999** |  |  |  |
| **1998** |  |  |  |
| **1997** |  |  |  |
| **1996** |  |  |  |
| **1995** |  |  |  |
| **1994** |  |  |  |
| **1993** |  |  |  |
| **1992** |  |  |  |
| **1991** |  |  |  |
| **1990** |  |  |  |
| **1989** |  |  |  |
| **1988** |  |  |  |
| **1987** |  |  |  |
| **1986** |  |  |  |
| **1985** |  |  |  |
| **1984** |  |  |  |
| **1983** |  |  |  |
| **1982** |  |  |  |
| **TOTALS** | **38223** | **626** | **6** |

**Supplement Table S3. Total counts from NCBI’s GenBank non-redundant nucleotide database with multiple sequencing technologies listed per entry.** Blank fields indicate absence of entries for the corresponding category.

Sequencing Technologies, Seq. Techs.

|  | **454** | **Illumina** | **IonTorrent** | **PacBio** | **SOLiD** |  |
| --- | --- | --- | --- | --- | --- | --- |
| **454** |  | **16** |  |  |  | **IonTorrent** |
| **454** |  | **3** |  |  |  | **PacBio** |
| **454** |  | **528** | **21** |  | **1** | **Sanger** |
| **Illumina** | **6** |  | **50** | **4** | **1** | **Sanger** |
| **Illumina** |  |  | **2** |  |  | **Oxford Nanopore** |
|  | **IonTorrent** |  |  |  |  |  |

**Supplement Table S4. Total counts from NCBI’s GenBank non-redundant nucleotide database of all entries with three and four sequencing technologies listed**

For example, there are a total of 6 entries in GenBank that have the following sequencing technologies listed: 454, Illumina, Ion Torrent, and Sanger for one sequence technology entry.

Pacific Biosciences, PacBio

| **Assembly** | **Year** | | | | | | | |
| --- | --- | --- | --- | --- | --- | --- | --- | --- |
| **Methods** | **2019** | **2018** | **2017** | **2016** | **2015** | **2014** | **2013** | **2012** |
| **ABySS** | 60 | 107 | 522 | 155 | 100 | 66 | 56 | 0 |
| **Bowtie** | 0 | 3 | 40 | 868 | 33 | 527 | 5 | 4 |
| **Bowtie2** | 2357 | 700 | 1682 | 128 | 787 | 9 | 51 | 0 |
| **BWA** | 430 | 856 | 671 | 294 | 281 | 440 | 148 | 1 |
| **Canu** | 9 | 29 | 3 | 0 | 0 | 0 | 0 | 0 |
| **Cap3** | 17 | 49 | 59 | 34 | 55 | 288 | 10 | 0 |
| **CLC** | 3364 | 3946 | 3404 | 5139 | 1948 | 2186 | 1172 | 381 |
| **DNA Baser** | 8 | 84 | 838 | 326 | 247 | 261 | 27 | 9 |
| **DNASTAR** | 2845 | 1953 | 4030 | 3191 | 6897 | 3175 | 3101 | 530 |
| **Geneious** | 1691 | 2764 | 3636 | 2633 | 4767 | 588 | 504 | 79 |
| **IDBA** | 452 | 259 | 28 | 11 | 729 | 22 | 2 | 0 |
| **MIRA** | 437 | 548 | 446 | 406 | 70 | 140 | 24 | 14 |
| **Newbler** | 9 | 295 | 176 | 183 | 703 | 336 | 435 | 60 |
| **Sequencher** | 137 | 425 | 3243 | 2154 | 2572 | 5727 | 7927 | 3462 |
| **SOAPdenovo** | 13 | 104 | 258 | 67 | 105 | 24 | 9 | 1 |
| **SPAdes** | 1736 | 2176 | 792 | 1632 | 89 | 266 | 0 | 0 |
| **Trinity** | 2274 | 1891 | 2162 | 4576 | 301 | 509 | 4 | 0 |
| **Velvet** | 85 | 258 | 161 | 107 | 338 | 341 | 144 | 32 |
| **Other** | 2839 | 3264 | 4190 | 6220 | 5810 | 5437 | 3179 | 6950 |
| **TOTALS** | **18763** | **19711** | **26341** | **28124** | **25832** | **20342** | **16798** | **11523** |

**Supplement Table S5. Total count of assembly programs used to generate sequences >2000 nt in the NCBI GenBank non-redundant nucleotide database.** These numbers were found with the following search criteria: “viruses,” “genomic RNA/DNA,” “GenBank (No RefSeq),” length: 2000 to 2000000, release date: 1/1/201X to 12/31/201X, and ‘”sequencing technology” in any field; the assembly method was then parsed out.

| **DBG** | | **OLC** | | **Proprietary Algorithm** | |
| --- | --- | --- | --- | --- | --- |
| **Program** | **Version** | **Program** | **Version** | **Program** | **Version** |
| ABySS | 2.0.2 | Cap | 3 | CLC Genomic Workbench | 11 |
| IDBA | 1.1.3 | Mira | 4.0.2 | Geneious | 10.2.3 |
| MetaSPAdes | 3.9.0 |  |  |  |  |
| SOAPdenovo2 | r240 |  |  |  |  |
| SPAdes | 3.9.0 |  |  |  |  |
| Trinity | 2.1.1 |  |  |  |  |

**Supplement Table S6. The 10 *de novo* assemblers used for analysis of the simulated data, as categorized by their underlying assembly algorithms.** de Bruijn graph, DBG; overlap-layout-consensus, OLC.
